# Task-Irrelevant Expectation Violations in Sequential Manual Actions: Evidence for a “Check-after-Surprise” Mode of Visual Attention and Eye-Hand Decoupling

**DOI:** 10.3389/fpsyg.2016.01845

**Published:** 2016-11-23

**Authors:** Rebecca M. Foerster

**Affiliations:** Neuro-cognitive Psychology, Department of Psychology & Cluster of Excellence Cognitive Interaction Technology ‘CITEC’, Bielefeld UniversityBielefeld, Germany

**Keywords:** eye movements, attention, expectation violation, surprise, manual action sequence, sensorimotor learning, eye-hand coupling

## Abstract

When performing sequential manual actions (e.g., cooking), visual information is prioritized according to the task determining where and when to attend, look, and act. In well-practiced sequential actions, long-term memory (LTM)-based expectations specify which action targets might be found where and when. We have previously demonstrated (Foerster and Schneider, 2015b) that violations of such expectations that are task-relevant (e.g., target location change) cause a regression from a memory-based mode of attentional selection to visual search. How might task-irrelevant expectation violations in such well-practiced sequential manual actions modify attentional selection? This question was investigated by a computerized version of the number-connection test. Participants clicked on nine spatially distributed numbered target circles in ascending order while eye movements were recorded as proxy for covert attention. Target’s visual features and locations stayed constant for 65 prechange-trials, allowing practicing the manual action sequence. Consecutively, a task-irrelevant expectation violation occurred and stayed for 20 change-trials. Specifically, action target number 4 appeared in a different font. In 15 reversion-trials, number 4 returned to the original font. During the first task-irrelevant change trial, manual clicking was slower and eye scanpaths were larger and contained more fixations. The additional fixations were mainly checking fixations on the changed target while acting on later targets. Whereas the eyes repeatedly revisited the task-irrelevant change, cursor-paths remained completely unaffected. Effects lasted for 2–3 change trials and did not reappear during reversion. In conclusion, an unexpected task-irrelevant change on a task-defining feature of a well-practiced manual sequence leads to eye-hand decoupling and a “check-after-surprise” mode of attentional selection.

## Introduction

When performing a manual action sequence in an unfamiliar environment (e.g., making a cup of tea in a hotel room), we have to search visually for the objects needed to perform the task (Ballard et al., 1992; Epelboim et al., 1995; Foerster et al., 2011; Foerster and Schneider, 2015b). In contrast, when acting in a familiar context, LTM can directly control gaze shifts to consecutive target objects in sequence, especially if the performed task is well-practiced. (Epelboim et al., 1995; Foerster et al., 2011, 2012; Foerster and Schneider, 2015b). As each of these task-driven gaze shifts is obligatorily preceded by a covert shift of attention (Deubel and Schneider, 1996), LTM controls for a sequence of attention and gaze shifts in this case. LTM-based attention and gaze control can be acquired through practice because sensorimotor routine tasks typically consist of fixed task elements that are repeated in a constant environment (e.g., making a cup of tea in your home kitchen). In this case, the sequence of perceptual input as well as of motor actions can be learned and automatized (see Robertson, 2007; Schuck et al., 2012; Schwarb and Schumacher, 2012 for perceptual vs. motor aspects of sequence learning and for the question whether sequences are learned on an item-to-item basis). However, sometimes sensorimotor routines have to be adapted to changing task elements or environments. In this case, the LTM-based mode of covert and overt (saccade) attentional selection has to be modified.

How is attentional selection modified if LTM-based expectations about probable object locations are no longer valid? If target objects are no longer at expected locations, visual search has to be reinitiated. Interestingly, if only a few target objects within a manual action sequence are unexpectedly displaced, visual search is performed even while having to act on unchanged targets in the sequence (Foerster and Schneider, 2015b). In Foerster and Schneider (2015b), participants had to click on eight numbered shapes in ascending sequence on a computer screen while eye movements were recorded. After having worked on a constant target position arrangement for 60-prechange trials, numbers 3 and 6 switched position. This action-sequence affecting change caused searching fixations while acting on the new located numbers, but also while acting on the consecutive non-displaced number 4. Eye-cursor coordination was even disturbed while acting on nearly any later target. These results imply that it is not possible to switch instantaneously back to the LTM-based mode of attention once it has been disturbed, even if this would be efficient for motor control. Instead, spatial changes that influence sub-actions of a sensorimotor action sequence cause a regression from an LTM-based mode of attentional selection to visual search beyond the change-affected sub-actions. In line with this result, further studies have shown that humans prefer visual information over memory information for action control in case of little automatization or a requirement for flexible behavior (Droll and Hayhoe, 2007; Patsenko and Altmann, 2010). However, while we have to adapt the mode of selection and manual action to target location changes in the environment, unexpected but action-irrelevant changes in target appearance do not necessarily afford a modification in selection and behavior. Nevertheless, processing such violations to LTM-based expectations about the task material might nevertheless have effects on covert and overt spatial attention allocation as well as manual action control, e.g., due to surprise (Horstmann and Herwig, 2015; Horstmann et al., 2016).

In Foerster and Schneider (2015b), expectation-discrepant shape changes of action targets (switch of shapes surrounding numbers 3 and 6) did neither affect eye movements, nor cursor performance arguing that LTM-based attentional selection was not disturbed by the action-irrelevant change. However, other studies have shown that non-spatial expectation-discrepant feature changes capture attention (Schützwohl, 1998; Horstmann, 2002, 2005). When a distractor has an unexpected feature, responding to the target slows down (Schützwohl, 1998) arguing that the expectation-discrepant distractor captures attention. Even if the target instead of a distractor appears with an expectation-discrepant feature, response slowing is often found (Horstmann, 2002, 2005, 2015). It has been argued that attention is allocated to the task-irrelevant surprising feature of the target instead to the feature that has to be reported (Horstmann, 2015). In line with this idea, gaze latency to a target with an unexpected color is shorter than to a target with an expected color, and fixations dwell longer on the first than on the latter (Horstmann and Herwig, 2015). An expectation-discrepant non-spatial feature seems to capture the eyes fast and binds attention thereafter – oculomotor capture. In real-world scenes, scene-inconsistent or otherwise expectation-discrepant objects are not only longer fixated, but also more frequently revisited (Loftus and Mackworth, 1978; Hollingworth and Henderson, 2002; Võ and Henderson, 2009; Võ et al., 2010) – a kind of second-order oculomotor capture. It seems that the surprising feature is rechecked repeatedly after having noticed it for the first time – a check-after-surprise mode of attentional selection.

Why is a check-after-surprise mode of attentional selection frequently applied during visual search (Loftus and Mackworth, 1978; Võ and Henderson, 2009; Võ et al., 2010; Horstmann, 2015; Horstmann and Herwig, 2015), but has not been found during sensorimotor control (Foerster and Schneider, 2015b)? In visual search, attention allocation is sensory-based, i.e., all visual objects and their features are potentially important to solve the task because the target can be anywhere in the visual environment. When performing a specific well-practiced sequential sensorimotor task, however, target features and locations are typically constant, so that LTM determines where-to-attend and where-to-look in sequence. Task-irrelevant objects and features are usually very effectively ignored (Land et al., 1999; Land and Hayhoe, 2001; Hayhoe et al., 2003; Droll et al., 2005; Foerster et al., 2011; Belardinelli et al., 2015). Thus, an expectation-discrepant but task-irrelevant feature seems to be effectively ignored in such tasks.

However, there are reasons to believe that a check-after-surprise mode of attentional selection could be useful during sequential sensorimotor control. In such tasks, changes of any kind might signal an unpredictable environment. Moreover, features without relevance in a specific task might become relevant for another related task. When walking a well-known route for shopping, it would be beneficial if attention would be captured by a road closure taking part behind, so that the way back can be planned efficiently. In summary, there is experimental evidence and arguments that speak against as well as in favor of adopting a check-after-surprise mode of attentional selection after task-irrelevant changes during sequential sensorimotor control.

A criterion that might determine whether task-irrelevant changes are noticed and modify attention is their relationship to the task-relevant objects of the task. In Foerster and Schneider (2015b), the target shapes were neither action-defining, nor in any other respect relevant throughout the experiment. Although, the eight individual shapes were obligatorily connected to the eight action-defining target numbers, the sensorimotor sequence could have been learned and executed equally well without the redundant shape information. Therefore, the shapes in the number-clicking task had no informational value for sensorimotor task control and could be completely ignored from the very first trial on. Correspondingly, the shape changes did not capture attention. However, a task-irrelevant change should be processed if it is related to an action-defining feature such as the appearance of a sign instructing your behavior (e.g., different looking traffic signs in a foreign country). Such task-irrelevant expectation violations might therefore initiate a check-after-surprise mode of attentional selection also during sensorimotor control.

Here, it was investigated whether and how a check-after-surprise mode of attentional selection is applied in a well-practiced manual sequence after a task-irrelevant change that is bound to an action-defining feature. In a computerized version of the number-connection test, participants had to click as fast as possible with a mouse cursor in ascending sequence on nine spatially distributed numbered circles on a computer screen. Eye movements were recorded as proxy for attentional selection based on the fact that a covert shift of attention obligatorily precedes every saccade (Deubel and Schneider, 1996). To ensure that an LTM-based mode of attentional selection was used prior to the introduced change, participants had to work on a constant configuration of numbered circles throughout 65 prechange-trials. In 20 successive change-trials, the task-irrelevant font of number 4 was changed. In 15 final reversion-trials, the originally presented and learned font was used again. The hypothesis is that the font change on the number is processed because the identity of the number is action-defining, as it specifies the position of the action target in the sequence – and had to be attended to learn the sensorimotor sequence. Thus, participants should be surprised and check for the new appearance of the number 4 after having noticed the font change. The aim of the study was to reveal at which moment within the sensorimotor sequence attention is captured by the expectation-discrepant number font and for how long it is revisited within the sensorimotor sequence as well as across several trials. Is the change noticed when having to act on the changed number or already when acting on prior targets in the sequence? Will the changed target 4 be checked only while having to act on it or also after having clicked on it successfully? Will the new appearance of the target elicit checking fixations even in subsequent trials? How fast can the reversion to the originally learned display with the originally learned fonts for all number be processed? Are eye movements and manual actions affected differentially by the change? These questions are important to understand how covert attention, gaze, and manual sequences are planned, preprogrammed, executed, and updated during sensorimotor control of well-practiced sequential manual actions.

## Materials and Methods

### Participants

Twenty right-handed students (8 males and 12 females, average age 25 years) from Bielefeld University, Germany, participated in the study after having provided written informed consent. All participants reported normal visual acuity, were naïve with respect to the purpose of the study, and were paid for their participation. The study was approved by the Committee for Ethics at Bielefeld University (EUB) and performed in accordance with the approved guidelines.

### Apparatus and Stimuli

The experiment took place in a dimply lit room and stimuli were displayed on a 19-inch color CRT monitor (ViewSonic Graphics Series G90fB using an ATI Radeon HD 2400 Pro graphics card) with a refresh rate of 100 Hz and a spatial resolution of 1024 pixels × 768 pixels extending 36 cm × 27 cm. Viewing distance was fixed with a chin-and-forehead rest at 71 cm. The experiment was controlled by the Experiment Builder software (SR Research, Ottawa, ON, Canada) on a Dell Optiplex 755 computer. The right gaze position was recorded with 1000 Hz by an EyeLink 1000 tower-mounted eye tracker (SR Research, Ottawa, ON, Canada). The computer mouse and keyboard were used as well as an extra-large mouse pad (32 cm × 88 cm). Color and luminance were measured in CIE Lxy coordinates using an X-Rite i1 Pro spectrophotometer.

All stimuli were displayed on a gray background (RGB 204, 204, 204; *L* = 78.9 cd/m^2^, *x* = 0.29, *y* = 0.30). The mouse cursor was a black dot of 0.43 degrees of visual angle (°v.a.) in diameter (RGB 0, 0, 0; *L* = 0.3 cd/m^2^, *x* = 0.32, *y* = 0.33). The target stimuli consisted of nine black numbered circles (circle diameter of 2.04°v.a.; bold type Arial numbers of font size 35 which equals to app. 0.96°v.a. height and 0.62°v.a. width, number 4 also in bold type MV Boli in some trials, color and luminance identical to the cursor). Circle number 1 was centered on the computer screen. The spatial layout of the remaining eight numbered circles was designed by randomly choosing locations within the outer fields of an imagined 3 × 3 grid with the prerequisite that the circles had a minimal distance of 2.04° v.a. to each other (border-to-border) as well as to the screen border. The spatial layout of the nine numbered circles was constant throughout the experiment.

### Procedure

**FIGURE 1:**
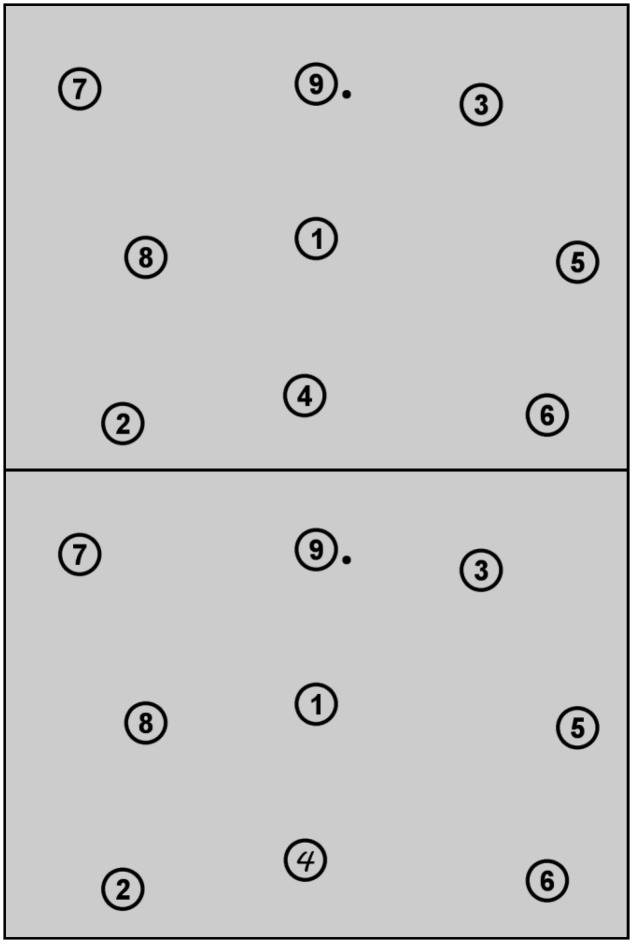
**Display during the clicking task in the prechange (top), the change (bottom), and the reversion (top) phase.** The black dot near number nine displays the mouse cursor.

Participants first read the instruction on the computer screen. They were asked to click on nine numbered circles in ascending order as fast as possible. A nine-point eye-tracking calibration and validation procedure followed. Only calibrations with an averaged accuracy below 1.0°v.a. were accepted. The first trial was announced as an example trial and thus not included in the analyses. The experiment consisted of a 65-trials prechange-acquisition phase (example trial excluded), a 20-trials change phase, and a 15-trials reversion phase. While the font of number 4 was Arial throughout prechange and reversion phase, it appeared in the font MV Boli throughout the change trials (**Figure 1**). All other numbers were displayed in Arial font throughout the experiment. A click was counted as correct within a diameter of 3.06°v.a. around a target’s center. A correct click was followed by a high-pitched tone. After all nine targets had been clicked in the correct sequence, a feedback display signaled the trial-completion time. A calibration check preceded each trial via a central fixation on a black ring (0.45°v.a. outer size, 0.11 °v.a. inner size). Calibration was repeated if necessary. After every block of 11 trials, a display informed participants about the number of completed and total experimental blocks. Participants started each block and trial by pressing the space bar. After having finished the last experimental trial, participants were asked whether they had noticed something peculiar. They used the keyboard to type in their answer. Subsequently, they were informed that indeed something was peculiar in the experiment and were asked to indicate which of 10 numbered statements did apply to the experiment (statements can be seen in **Table 1**). Selection was performed by typing in the selected statement numbers. All participants completed the experiment within 40 min. The participant with the fastest best time earned 2€ extra.

**Table 1:** The English translation of the 10 numbered statements participants could indicate as applicable to the experiment as well as the number of choices (right column).

(0) The size of one or several stimuli was changed.	0
(1) One had to click spatially displaced on one or several stimuli.	1
(2) The order was changed.	3
(3) The font of one or several numbers was changed.	18
(4) One or several stimuli were spatially displaced.	3
(5) The shape of one or several stimuli was changed.	0
(6) The size of one or several numbers was changed.	2
(7) Clicks were not always accepted.	2
(8) One or several numbers was spatially displaced.	2
(9) One or several numbers were missing.	0

### Analysis

The following dependent variables were analyzed: Trial-completion times, number of errors and fixations, scanpath and cursor-path lengths, as well as eye–cursor distance. The SR Research EyeLink Data Viewer software’s implemented default velocity algorithm was used to detect fixations (not a blink, <30°v.a./s velocity and <8,000°v.a./s^2^ acceleration). Scanpath and cursor-path lengths were calculated as 100-Hz cumulative inter-sample distances. Eye–cursor distance was calculated as 100-Hz intra-sample distance.

To reveal whether LTM-based attentional control was built up over the course of the prechange phase, analyses of variance (ANOVAs) studied the state of learning through the first five prechange blocks (1–11, 12–22, 23–33, 34–44, and 45–55). To analyze the effects of the font change, paired *t*-tests were conducted to compare the very first change trial (trial 66) to the prechange baseline consisting of the average of the last ten prechange trials (56–65). For fine-grained analyses, further within-subject variables were sub-action (1–9), location (1–9), and fixation type (searching, guiding, and checking). Fixation types were defined according to their landing positions (Epelboim et al., 1995; Land and Tatler, 2009; Foerster and Schneider, 2015a,b): fixations on any upcoming target (except the current target) as searching, fixations on a current target as guiding, and fixations on any completed target as checking (interest area of 3.06°v.a. diameter). To analyze how long the effects of the changed font might last when repeating the changed display, change trials 2–5 were also compared to prechange baseline with paired *t*-tests. To reveal whether the reversion to the originally learned display had any effects on performance and gaze control, paired *t*-tests were used to compare the very first reversion trial to the prechange baseline. Violations of sphericity were corrected by using the Greenhouse-Geisser 𝜀 (uncorrected degrees of freedom are provided to facilitate reading). A chance level of 0.05 was applied.

## Results

This section is divided into four parts. First, it is analyzed whether participants adopted an LTM-based attention mode over the course of the prechange phase (five blocks). Second, I report the effects of the unexpected font change on manual performance and eye movements to reveal whether there was a shift in the applied mode of attentional selection, i.e., from an LTM-based mode to a check-after-surprise mode of attentional selection. Third, I report the effects on attentional control by several repetitions of the changed display as well as by the reversion to the originally learned display. The third investigation will reveal how long the surprising font change affected manual and eye movement parameters before an LTM-based mode of attentional control was reinitiated as well as whether the reversion to the prior font was as surprising in terms of modifications of gaze and manual action parameters as the initial font change. Finally, the answers to the explicit awareness questions will be summarized.

### Prechange Phase: Acquisition of a LTM-Based Mode of Attentional Selection

**FIGURE 2:**
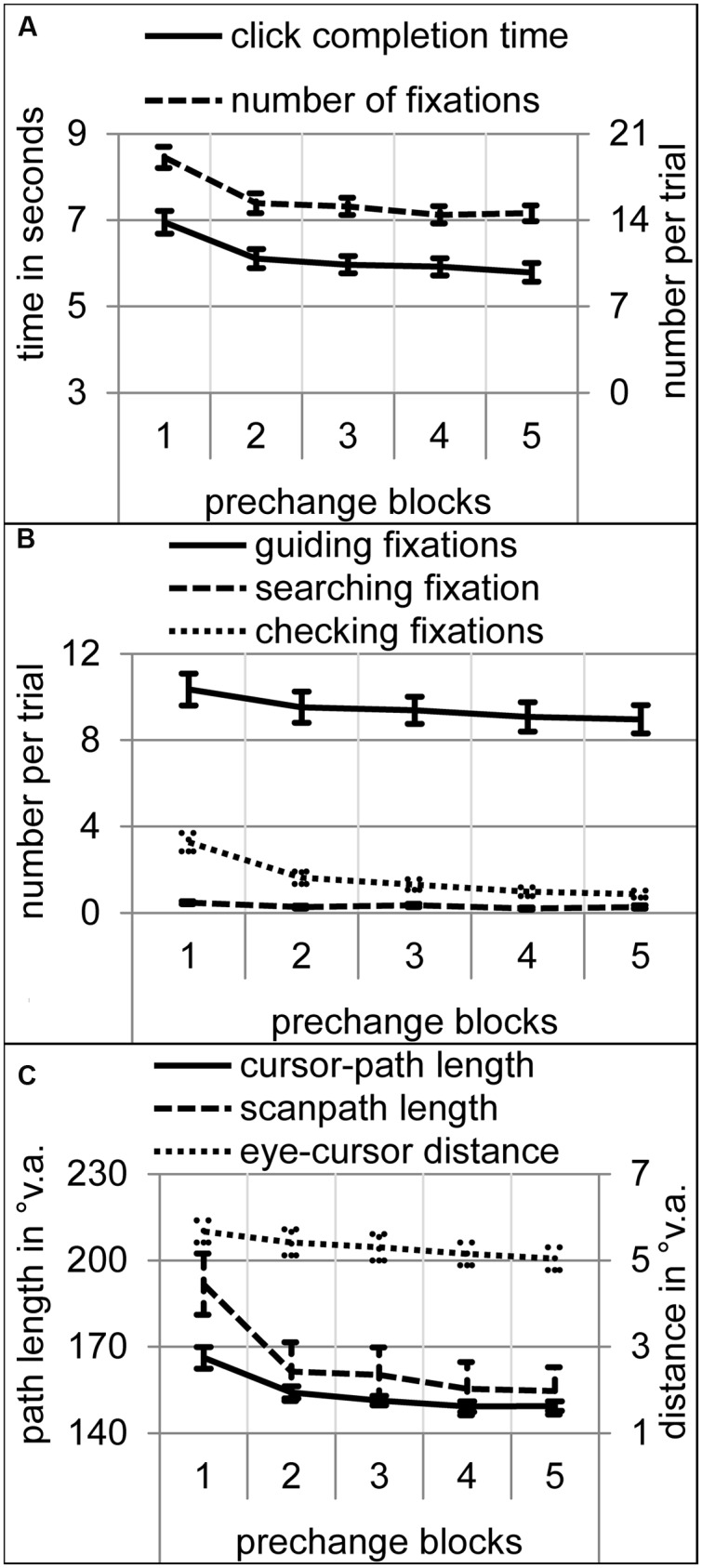
**Performance and eye movement measures over the course of the five prechange blocks.** Error bars represent standard error of the means. **(A)** Click completion time in seconds and number of fixations per trial. **(B)** Number of searching, guiding, and checking fixations per trial. **(C)** Cursor-path and scanpath length as well as eye-cursor distance in °v.a.

Did participants adopt an LTM-based mode of attentional selection within the prechange phase? Over the course of the first five prechange blocks, trial completion time, number of fixations, cursor-path and scanpath length, and eye-cursor distance decreased as is typical for sensorimotor learning [**Figures 2A,B**; time: *F*(4,76) = 48.38, *p* < 0.001; linear trend *F*(1,19) = 75.63, *p* < 0.001; fixations: *F*(4,76) = 41.99, 𝜀 = 0.56, *p* < 0.001; linear trend *F*(1,19) = 71.42, *p* < 0.001; cursor-path: *F*(4,76) = 23.28, 𝜀 = 0.53, *p* < 0.001; linear trend *F*(1,19) = 33.07, *p* < 0.001; scanpath: *F*(4,76) = 30.00, 𝜀 = 0.48, *p* < 0.001; linear trend *F*(1,19) = 48.60, *p* < 0.001; eye-cursor distance: *F*(4,76) = 6.60, 𝜀 = 0.53, *p* < 0.01; linear trend *F*(1,19) = 13.11, *p* < 0.01]. An ANOVA on the number of fixations with block and fixation type as within-subject variables revealed significant main effects of block and type as well as a significant interaction [block: *F*(4,76) = 29.15, 𝜀 = 0.54, *p* < 0.001; type: *F*(2,38) = 158.39, 𝜀 = 0.62, *p* < 0.001; block by type: *F*(8,152) = 4.92, 𝜀 = 0.27, *p* < 0.05]. All types of fixations decreased significantly in the course of the prechange phase [**Figure 2C**; searching fixations: *F*(4,76) = 4.10, 𝜀 = 0.64, *p* < 0.05, linear trend *F*(1,19) = 7.31, *p* < 0.05; guiding fixations: *F*(4,76) = 3.75, 𝜀 = 0.46, *p* < 0.05, linear trend *F*(1,19) = 4.90, *p* < 0.05; checking fixations: *F*(4,76) = 33.40, 𝜀 = 0.41, *p* < 0.001, linear trend *F*(1,19) = 45.14, *p* < 0.001]. On average, significantly more guiding fixations were performed than searching and checking fixations, and more checking than searching fixations (all *p*s < 0.001). During the fifth prechange block, participants performed on average 8.95 guiding, 0.87 checking, and 0.27 searching fixations per trial. Guiding the hand (here cursor) sequentially with approximately one fixation to each target on an effective path is a typical characteristic of LTM-based attentional selection for sensorimotor control (Foerster et al., 2011, 2012; Foerster and Schneider, 2015a,b). None of the dependent variables was significantly different across blocks 4 and 5. Thus, a first plateau of gaze and manual action performance seemed to be reached after the 4th block.

### First Task-Irrelevant Change Trial: Shift to a Checking-after-Surprise Mode of Attentional Selection

**FIGURE 3:**
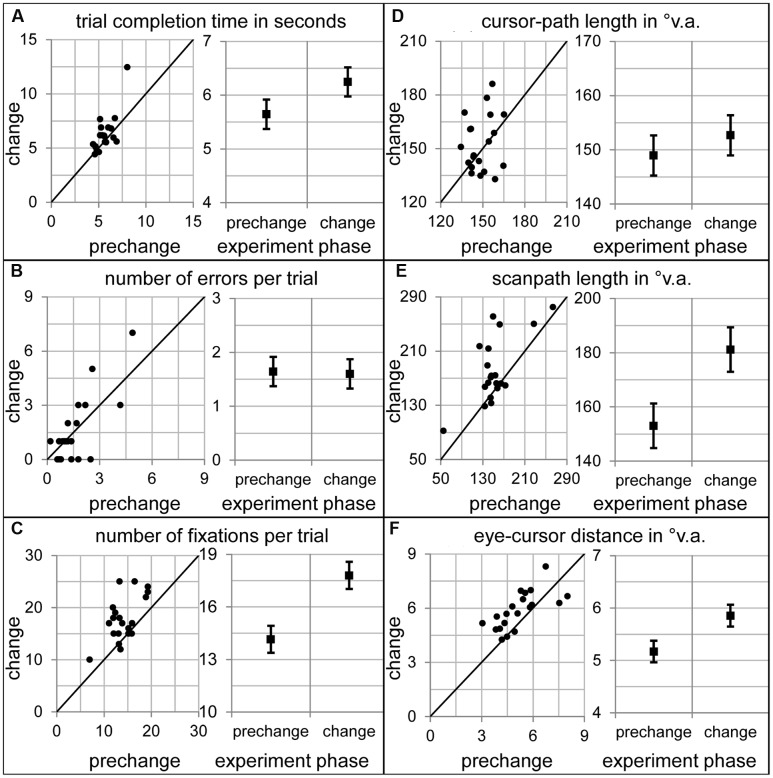
**Dependent variables during prechange baseline and during the first change trial.** The left diagrams of all panels show the 20 participants’ individual data. The right diagrams of all panels show the sample means and the standard error of the paired mean differences. **(A)** Click completion time in seconds. **(B)** Number of errors per trial. **(C)** Number of fixations per trial. **(D)** Cursor-path length in °v.a. **(E)** Scanpath length in °v.a. **(F)** Eye-cursor distance in °v.a.

How did participants allocate their overt attention within the sensorimotor sequence, when number 4 appeared unexpectedly in another font? To answer this question, the dependent variables of the prechange baseline (last ten prechange trials) were compared to the very first change trial (**Figure 3**). The completion time of the change trial was significantly longer than in the prechange baseline [*t*(19) = 2.20, *p* < 0.05, **Figure 3A**]. In addition, participants performed more fixations [*t*(19) = 4.72, *p* < 0.001, **Figure 3C**] during the change trial. Number of errors and cursor-path length was not significantly affected by the font change [*t*(19) = 0.17, *p* = 0.87, **Figure 3B** and *t*(19) = 1.00, *p* = 0.33, **Figure 3D**, respectively]. However, scanpaths length and eye-cursor distance was larger when acting on the changed than the learned prechange display [*t*(19) = 3.43, *p* < 0.01, **Figure 3E**, and *t*(19) = 3.31, *p* < 0.01, **Figure 3F**, respectively].

**FIGURE 4:**
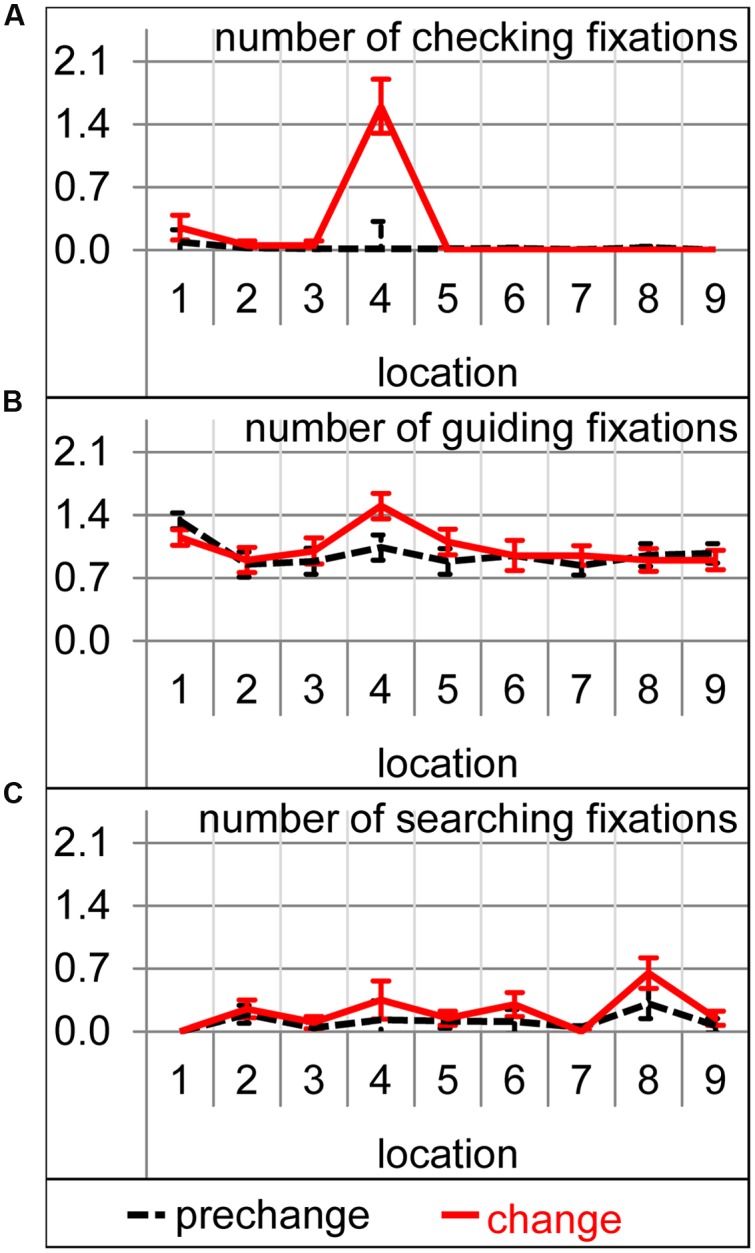
**Number of the checking (A),** guiding **(B)**, and searching **(C)** fixation per trial on each of the nine target locations during prechange baseline (broken black lines) and the first change trial (solid red lines). Error bars represent standard errors of the paired difference between prechange and change for each location.

An analysis of the number of the different fixation types revealed that significantly more searching and checking fixations were performed during the change trial [*t*(19) = 2.68, *p* < 0.05 and *t*(19) = 4.28, *p* < 0.001, respectively], but not significantly more guiding fixations [*t*(19) = 1.41, *p* = 0.17]. For the number of checking fixations, the interaction between condition (prechange vs. change) and location (1–9) was significant [*F*(8,152) = 22.48, 𝜀 = 0.18, *p* < 0.001]. The additional checking fixations were exclusively directed to number 4 [*t*(19) = 5.24, *p* < 0.001, **Figure 4A**]. Obviously, the changed font of number 4 caused attentional and oculomotor revisiting. Also for the number of guiding fixations, the condition by location interaction reached significance [*F*(8,152) = 2.11, 𝜀 = 0.83, *p* < 0.05]. More guiding fixations were performed on the changed number 4 [*t*(19) = 3.26, *p* < 0.01, **Figure 4B**]. The increase in searching fixations was not accompanied by a significant condition-by-location interaction [*F*(8,152) = 1.15, 𝜀 = 0.43, *p* = 0.34]. Thus, the increase in searching fixations was not concerned with a specific location (**Figure 4C**).

**FIGURE 5:**
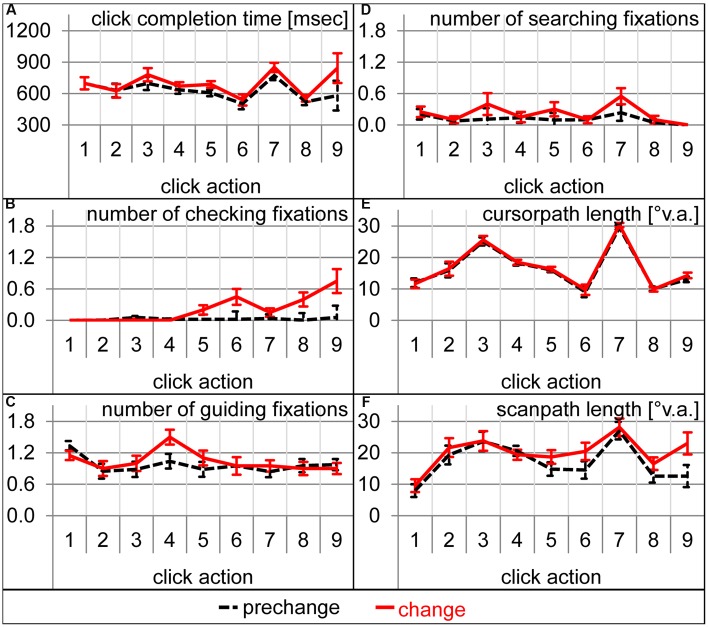
**Dependent variables during each of the nine clicking actions during prechange baseline (broken black lines) and the first change trial (solid red lines).** Error bars represent standard errors of the paired difference between prechange and change for each click action. **(A)** Click completion time in milliseconds. **(B)** Number of checking fixations. **(C)** Number of guiding fixations. **(D)** Number of searching fixations. **(E)** Cursor-path length in °v.a. **(F)** Scanpath length in °v.a.

During which sub-action of the sensorimotor sequence was participants’ attention captured by the expectation-discrepant appearance of number 4? To answer this question, analyses of variance with click action (1–9) and condition (prechange vs. change) as within-subject factors were calculated for the number of each fixation type. For the number of checking fixations, the interaction between condition and click action was significant [*F*(8,152) = 5.98, 𝜀 = 0.39, *p* < 0.01] as were both main effects [condition: *F*(1,19) = 18.31, *p* < 0.001; action: *F*(8,152) = 6.29, 𝜀 = 0.40, *p* < 0.01]. Significantly more checking fixations were performed during click actions 6 [*t*(19) = 2.83, *p* < 0.05], 8 [*t*(19) = 2.93, *p* < 0.01], and 9 [*t*(19) = 3.05, *p* < 0.01], and marginally during click action 5 [*t*(19) = 2.03, *p* = 0.06; **Figure 5B**]. The analysis of guiding fixations per click action is identical to the analysis of guiding fixation per location, as guiding fixations are always concerned with the current action target location. As already mentioned above, the changed font caused more guiding fixations for number 4 and thus also during click action 4 (**Figures 4B** and **5C**). The analysis of the number of searching fixations resulted in no significant interaction between condition and click action [*F*(8,152) = 1.24, 𝜀 = 0.46, *p* = 0.30], but a significant main effects of condition [*F*(1,19) = 7.20, *p* < 0.05] and action [*F*(8,152) = 3.72, 𝜀 = 0.47, *p* < 0.01). Thus, some sub-actions afforded generally more searching than others. However, the increase in the searching behavior was not concerned with a specific sub-action of the sensorimotor sequence. Also for scanpaths length, the interaction of condition and click action did not reach significance [*F*(8,152) = 1.24, 𝜀 = 0.46, *p* = 0.30], but the main effects of condition [*F*(1,19) = 11.76, *p* < 0.01, **Figure 5E**] and action [*F*(8,152) = 11.45, 𝜀 = 0.50, *p* < 0.001] did. Scanpaths were generally longer during some click actions, and prolonged due to the change. However, their prolongation was not concerned with a specific click action. While eye movement parameters were strongly affected by the font change, manual performance did not suffer remarkably. Trial completion time was prolonged (see above). The increase in completion time was not concerned with a specific click action [action by condition interaction: *F*(8,152) = 1.63, 𝜀 = 0.38, *p* = 0.19, **Figure 5A**]. Cursor-path length did not at all increase as already mentioned above (**Figure 3D**, also **Figure 5D** per click action). This dissociation between eye and cursor movements was confirmed by the increased distance between eye and cursor. Not only the main effects of condition [*F*(1,19) = 10.96, *p* < 0.01] and action [*F*(8,152) = 18.86, 𝜀 = 0.52, *p* < 0.001], but also the interaction was significant [*F*(8,152) = 3.35, 𝜀 = 0.53, *p* < 0.05]. Eye-cursor distance was increased across click actions 6–9 [6: *t*(19) = 2.81, *p* < 0.05; 7: *t*(19) = 2.19, *p* < 0.05; 8: *t*(19) = 2.64, *p* < 0.05; 9: *t*(19) = 3.58, *p* < 0.01, **Figure 5F**]. Thus, eye-cursor coupling was disturbed after having acted on the font-changed number 4, but not before.

In summary, when the task-irrelevant font of the action-defining number 4 changed unexpectedly in the well-practiced sensorimotor task, the LTM-based mode of attentional selection was replaced by a check-after-surprise mode of attentional selection. Moreover, while the surprising feature frequently attracted the eyes after it had been acted on, the hand continued the task without much interference. This result revels that eye-hand coupling is loosened in the service of maintaining manual performance.

### Change Repetition and Reversal: Reinitiation of the LTM-Based Mode of Attentional Selection

The effects on attentional control by several repetitions of the changed display as well as by the reversion to the originally learned display can be found here. Investigating the effects elicited by a repetition of the font-changed display can reveal how long the unexpected font was rechecked with the eyes during task performance until an LTM-based mode of attentional control was reinitiated. Planned *t*-tests were performed to compare the four subsequent change trials (trials 67–70) individually to the prechange baseline. The first repetition of the changed display resulted in a longer trial completion time [*t*(19) = 2.31, *p* < 0.05], more fixations [*t*(19) = 5.20, *p* < 0.001], which were searching fixations [*t*(19) = 4.18, *p* < 0.001], longer scanpaths [*t*(19) = 3.31, *p* < 0.01], and a larger eye-cursor distance [*t*(19) = 2.75, *p* < 0.05]. More fixations were also performed during the second repetition of the changed display [*t*(19) = 2.54, *p* < 0.05], but this time more guiding fixations [*t*(19) = 3.00, *p* < 0.01]. All other dependent variables did no longer differ significantly to prechange baseline. Thus, the surprising deviant font did affect performance and gaze control only up to two repetitions before participants worked in a LTM-based mode of attentional control again.

Did the reversion to the previously learned Arial-font display elicit the same surprise effect as the initial font change? The very first reversion trial (86) differed significantly from the prechange baseline only in trial completion time [*t*(19) = 2.60, *p* < 0.05]. However, participants were not slower, but faster during the reversion trial, perhaps due to further motor refinement over the course of the change phase. Thus, the reversion to the originally learned display did not elicit any check-after-surprise effects in terms of gaze performance changes.

### Explicit Awareness of the Font Change

In order to reveal, whether participants were explicitly aware of the font change, they were asked after the experiment whether they had noticed something peculiar. Ten of the 20 participants spontaneously reported that the font of number 4 did change within the experiment. When participants had to select a noticed change from ten presented alternatives (see **Table 1**), 18 of the 20 participants selected the font change. Nine participants indicated further statements to be true. Most participants seemed to have noticed the font change explicitly. The occasional entry of further observed changes might have been encouraged by the permission of multiple selections and a natural suspicion of psychology students with respect to experimental manipulations.

## Discussion

In the present study, it was investigated whether and how attentional selection of action targets for sequential motor routines is modified when confronted with task-irrelevant expectation-violations. Although environmental changes that are not relevant for the current task do not require action modification, they are nevertheless unexpected and might therefore influence overt attentional selection and manual control. Especially if task-irrelevant aspects of action-defining features are changed, attentional selection based on LTM expectations might be disturbed. The hypothesis was that a task-irrelevant change on an action-defining feature of a target should lead to rechecking of the expectation-violating object. Revisits of the changed target could be purely oculomotor or also manual, which has different consequences for eye-hand coupling. It was investigated how long possible effects of the change would last within the action sequence as well as when repeatedly displaying the changed target.

In a computerized version of the number-connection test, participants clicked as fast as possible with a mouse cursor in ascending sequence on nine spatially distributed numbered circles on a computer screen, while gaze was recorded. Participants had to work on a constant configuration of numbered circles throughout 65 prechange-trials. In 20 successive change-trials, the font of number 4 changed. In 15 final reversion-trials, the originally learned font was used again. Results revealed that the font-changed number 4 captured attention and eye movements as soon as number 4 had to be acted on. Cursor movements, however, were not at all affected. The asymmetry between eye and cursor effects was reflected by an enlarged eye-cursor distance throughout the remaining trial. The effects lasted for up to two repetitions of the changed target display, but did not reoccur when reverting to the originally learned display.

In the following, the results are discussed with respect to the involvement of a sensory-based vs. memory-based control mode of attention and eye movements when performing well-practiced sequential sensorimotor actions. Afterward, the checking-after-surprise gaze effect will be dissociated from gaze effects caused by the need for a modification of a learned sensorimotor sequence. Finally, the limits of eye-hand coupling are discussed.

### Sensory-Based versus Memory-Based Control of Attention and Gaze

When having to determine where to attend next to achieve an ongoing task, different sources of information can be used. Sensory information is weighted according to its task-relevance (Bundesen et al., 2011; Bundesen and Habekost, 2014; Poth et al., 2014). Attention and gaze can then be shifted to the location containing the most relevant information (e.g., highest attentional weight) for the current task (Wischnewski et al., 2010; Schneider, 2013). Alternatively, task-related memory can be used to shift attention and gaze directly to a retrieved target position without the need to process visual features (Foerster et al., 2011, 2012, Jiang et al., 2013, 2014). Especially, when performing well-known sensorimotor actions, strong memory codes are used to direct attention and gaze in a task-dependent manner (Foerster et al., 2011, 2012). When switching on your bedside light in the dark, attention and gaze can be directly shifted to the light switch from LTM, allowing to perform the task even in complete darkness (cf. Foerster et al., 2012). Usually, both memory-based and sensory-based control of attention is applied to achieve a task.

Investigating which available sensory information is still modulating gaze control in a well-practiced sensorimotor task can reveal whether and how the contents of task sets are modulated throughout sensorimotor learning, such as relying more or even exclusively on LTM for action control and ignoring specific sensory information completely (Schneider and Shiffrin, 1977; Logan, 1988). In the present study, sensory-based attention and gaze control were still applied after extensive learning, even for constant sensory features that are no longer indicative for successful action control. The font change caused participants to frequently revisit the font-changed number 4, although the change did not afford a modification of the learned and memorized sensorimotor trajectory. Participants also noticed the font change explicitly. Conclusively, they did still process the sequence-defining identity of the numbers which is not separable from its font. Number identities were processed although LTM would have sufficed to determine and execute the clicking sequence. It seems that the task set was still defined according to the instruction to click the numbered circles in ascending sequence. Participants did not modulate the task set, e.g., into “click the learned sequence.” Participants still used sensory information for gaze control and did not rely completely on learned spatial or motor codes (see Hikosaka et al., 1999; Rand et al., 2000; Richard et al., 2009 for the application of spatial and motor codes in sensorimotor sequences). Thus, even in well-practiced sensorimotor tasks, the available sensory information is still extracted and processed according to explicit task sets. Cursor movements, however, were not affected by the changed sensory information. This finding argues for a stronger contribution of spatial and motor codes for manual than for oculomotor control.

The continued application of sensory-based gaze control is in line with the observation that the eyes typically sample sensory information for a current sub-action just in time (Land et al., 1999; Hayhoe et al., 2003; Gajewski and Henderson, 2005). The currently important visual features and locations are extracted shortly before they are needed even if they could be recalled from memory (Gajewski and Henderson, 2005; Droll and Hayhoe, 2007; Foerster et al., 2011, 2012). Using the world as external memory saves memory load (O’Regan, 1992). Moreover, the current visual information is more reliable and richer in detail than an error-prone memory trace (Gray and Fu, 2004). Finally, revisiting action-relevant visual information ensures fast and efficient adaptation to environmental changes across repetitions if required (Adam et al., 2012).

### Gaze Effects Caused by Surprise versus the Need for Sensorimotor Modification

In the present investigation, modification of the manual trajectory was not needed as the action-defining number identity was not changed, only its font. The font change detection did nevertheless affect gaze behavior. The changed gaze behavior in the present study does not reflect a consequence of the need for a sensorimotor modification in response to a trajectory change as was the case in Foerster and Schneider (2015b). Instead the changed oculomotor selection here represents a surprise reaction to the task-irrelevant expectation violation. There are three differences in the gaze effects caused by surprise vs. the need for sensorimotor modification.

First, when a detected change affords a modification of a well-practiced sensorimotor sequence, gaze control regresses from a LTM-based mode to a visual search mode (Foerster and Schneider, 2015b). In such a case, not yet completed action targets are frequently visited in order to find the changed target locations or sequence. However, completed action targets are still nearly completely ignored, i.e., no checking fixations are performed. When an unexpected change elicits a surprise effect, oculomotor capture is observed, i.e., the surprising feature is fixated longer and is frequently revisited (Loftus and Mackworth, 1978; Hollingworth and Henderson, 2002; Võ and Henderson, 2009; Võ et al., 2010). Correspondingly, in the present study, the font-changed number 4 was frequently checked after having successfully clicked on it. Interestingly, this modulation of attentional selection was not accompanied by a change in the manually controlled cursor trajectory. This result pattern can also preclude that the effects are due to a lack of priming for the new font. Primed stimuli can be selected and responded to faster than unprimed stimuli (Meeter and Van der Stigchel, 2013; Kruijne and Meeter, 2016). However, the increase in clicking time in the present study was not limited to the font-changed number. In addition, the cessation of priming for the font-changed number would not predict a revisiting of this unprimed material, especially not after having clicked on it successfully.

Second, the effects on attentional selection caused by the need to modify a well-practiced sensorimotor sequence remains for several repetitions. Up to 15 trials were needed to fully integrate a two-numbers location switch in the 8-target sequence of Foerster and Schneider (2015b), so that gaze control showed again all characteristics of LTM-based selection. Surprise effects, however, are typically short-lived. Only the very first presentation of a deviant stimulus style resulted in longer reaction times in the study of Schützwohl (1998), while reaction times to repeated deviant styles were not significantly different from prechange baseline. Also the gaze effects elicited by the font change of the present study did result in relatively short-lasting effects (1–3 trials).

Third, the reinitiation of a previously learned sensorimotor sequence is accompanied by the same visual search gaze behavior as the first modification (Foerster and Schneider, 2015b). Contrastingly, the reversion to a familiar visual presentation elicits usually no response change indicative for a surprise in contrast to the change from a familiar to an unfamiliar stimulation (e.g., Schützwohl, 1998; Horstmann, 2002; Horstmann and Herwig, 2015). Surprising a person twice is difficult enough, especially with something that is already known. Correspondingly, reversion to the original font in the present study did neither impair performance nor affect gaze. It is likely, that even if a new deviant had been introduced, no further or a far smaller surprise effect would have arisen. The introduction of a deviant should change the expectation about possible task elements “once and for all” as Gaschler et al. (2015) put it. On the basis of these three considerations, the gaze modification observed in the present study constitutes a check-after-surprise effect.

### The Limits of Eye-Hand Coupling

When performing a sensorimotor task, eye and hand movements are typically tightly coupled (Neggers and Bekkering, 2000; Beurze et al., 2009; Song and McPeek, 2009). Before the hand or a manipulated tool reaches a specific action target, gaze is shifted to the target position (Land et al., 1999; Hayhoe et al., 2003; Sailer et al., 2005; Foerster et al., 2011, 2012). Eye movements typically precede hand movements. First, visual information important for hand motor planning can be extracted, e.g., target size and its orientation (Prablanc et al., 1979, 1986, 2003; Prablanc and Martin, 1992; Paillard, 1996; Land et al., 1999; Crawford et al., 2004; Prado et al., 2005; Beurze et al., 2006). Second, even if the visual appearance of the target is known, motor performance should benefit from the well-learned eye to hand motor transformations (Gnadt et al., 1991; Henriques et al., 2003; Crawford et al., 2004; Flanagan et al., 2008). Third, gaze can possibly be used as deictic pointer for the eyes (Ballard et al., 1992, 1997; Neggers and Bekkering, 2001; Flanagan et al., 2008; Rosenbaum, 2010). Forth, fixating an action target might serve as retrieval cue for the required action on the target or upcoming subactions of the sensorimotor sequence (Laeng and Teodorescu, 2002; Johansson et al., 2011; Johansson and Johansson, 2013). However, eye and hand can be decoupled if explicitly required by the task. We can, for instance, simultaneously saccade to one location and reach to another. In this case, attention is allocated in parallel to the saccade and reaching target prior to motor initiation (Jonikaitis and Deubel, 2011). An unanswered research question is how eye and hand movements are selected during sensorimotor actions in which the eyes are not arbitrarily restricted but assist the manual actions as is typical in real-world situations. It is possible that a common mechanism selects eye and hand target positions in this case (Schneider, 1995; Deubel and Schneider, 2003). Alternatively, eye and hand target positions might nevertheless be selected by different attentional mechanisms. If the latter is the case, spontaneous decoupling of eye and hand movements could occur even if the task does not afford a decoupling. In the present study, such a decoupling of eye and hand movements was observed. While the eyes revisited the font-changed number frequently, the hand-controlled cursor proceeded to move sequentially and with a similar speed to the remaining action targets. However, each target click was still preceded by a target fixation. Thus, eye-hand coupling was only partly abandoned for the sake of performance maintenance. While, not every saccade target selection was coupled to a cursor target selection, every manual target selection (click) was preceded by a saccade on the selected target. This is a nice analogy to the well-known finding that attention can be shifted covertly without moving the eyes, while each saccade is obligatorily preceded by a covert shift of attention (Deubel and Schneider, 1996). Future studies have to identify whether the results might generalize to tasks with other requirements, e.g., higher accuracy requirements when acting on objects with varying shapes or real-world interactions with three-dimensional objects.

### Summary

In the present study, a task-irrelevant target feature change in a well-practiced sensorimotor task affected gaze behavior, while manual performance was hardly changed. Although target features can be retrieved from LTM in a well-practiced sensorimotor task, target features that are action-defining according to the task set seem to be still visually processed. That is why the font change of the action-defining number target was detected in the present study. Detecting such action-defining feature changes allows flexible sensorimotor adaptation whenever needed. Although the detected font change did not require a sensorimotor adaptation in the present study, the deviant-font number was frequently revisited by the eyes. The violation of the learned LTM prediction elicited a surprise resulting in a checking mode of attention and gaze control for up to two repetitions of the deviant target display. Manual performance was hardly affected demonstrating that eye and hand movements can be efficiently decoupled in order to maintain a high level of task performance. Nevertheless, eye-hand coupling was still preserved for all target clicks displayed by target fixations guiding all successful mouse clicks. Therefore, an LTM-based mode of attention and gaze control is combined with a check-after-surprise mode after detecting task-irrelevant target feature changes while performing a well-practiced sensorimotor sequence.

## Author Contributions

RF designed and programmed the experiment, performed the data analysis, interpreted and discussed the results, and wrote the manuscript.

## Conflict of Interest Statement

The author declares that the research was conducted in the absence of any commercial or financial relationships that could be construed as a potential conflict of interest.
